# Gender and household structure factors associated with maternal and child undernutrition in rural communities in Ethiopia

**DOI:** 10.1371/journal.pone.0203914

**Published:** 2018-10-04

**Authors:** Getahun Ersino, Gordon A. Zello, Carol J. Henry, Nigatu Regassa

**Affiliations:** 1 Nutrition and Dietetics, College of Pharmacy and Nutrition, University of Saskatchewan, Saskatoon, Saskatchewan, Canada; 2 School of Nutrition, Food Science and Technology, Hawassa University, Hawassa, SNNPR, Ethiopia; CUNY, UNITED STATES

## Abstract

Addressing maternal and child undernutrition is a priority for the National Nutrition Program of Ethiopia. In a cross-sectional design, we selected mother-child pairs (n = 630) from Halaba, south Ethiopia (n = 413, two communities) and Zeway, Oromiya region (n = 217, one community). These communities were previously included in a project to improve agricultural practices. We aimed to estimate the level of maternal and child undernutrition in the two study sites and compare findings to regional/national reports. We also examined associations with gender, household-structure and nutrition/health related variables. Households were selected using simple random sampling based on list of households obtained from local health posts. Mothers were interviewed via questionnaire. Anthropometric measurements were taken from mothers-child pairs. Maternal undernutrition (% BMI<18.5) ranged from moderate (14% Zeway) to high (22% Halaba). In the children, stunting and underweight were very high (54% and 42% stunting, 36% and 21% underweight, in Halaba and Zeway, respectively). Up to 95% of Halaba and 85% of Zeway mothers reported “same as usual” or “less than usual” consumption patterns during their most recent pregnancy compared to periods of non-pregnancy. Mothers reported (61% in Halaba, 18% in Zeway) abstaining from consumption of certain nutritious foods for cultural reasons. Gender and socio-economic-demographic structure of the households, including imbalance of power, control of farm produce, physiological density, household size and dietary habits during pregnancy showed significant associations with maternal and child undernutrition (*p*<0.05). The levels of child and maternal undernutrition, particularly in children, were unexpected and of concern, given that a national nutrition program has been in place since 2008. The study provides insights for policy makers to improve women’s education, reproductive health services for better family planning, and strengthen nutrition/health programs designed to target vulnerable segments of the population in these and other rural communities and districts with similar structure and demographics in Ethiopia.

## Introduction

Despite progress in the past 15 years, levels of child stunting and maternal undernutrition in Ethiopia remain unacceptably high [1]. The levels reported in the 2011 Demographic and Health Survey (DHS) showed slight improvement (in children only) compared with previous DHS [2], but were still *‘very high’* by World Health Organization (WHO) standard [3]. A mini-DHS survey in 2014 reported a 40.4% prevalence of child stunting in Ethiopia, a small improvement over a five year period [4]. Maternal undernutrition, however, had not changed since the first DHS in 2000 [1, 2, 5].

Maternal and child undernutrition has been a local and global public health concern with far-reaching consequences, ranging from poor health outcomes to loss of economic gains and productive citizens due to morbidity and mortality associated with poor nutrition. Research has estimated over 3 million child deaths annually (45% of all child deaths) due to undernutrition and suboptimal care for young children [6, 7]. Undernutrition in mothers has been identified as a major risk factor for poor health outcomes in offspring, such as poor fetal-growth, low-birthweight and poor development in the first two years after birth, resulting in stunted-growth and poor health later in life [6, 8, 9]. Child undernutrition particularly stunting is a major problem. Stunting is linked with some serious consequences such as poor cognitive development, lower school performance–hence, loss of income and productivity—which further perpetuates intergenerational undernutrition due to the subsequent poor economic capacity to provide adequate care for one’s offspring [10]. Realizing the grave consequences of malnutrition, local and international governments, as well as humanitarian agencies have sought ways to mitigate the problem of undernutrition through implementation of various health and nutrition programs.

Since 2008, the Ethiopian government has implemented a National Nutrition Program (NNP) that aimed at improving the nutrition of women and children [11]. The NNP set a specific target for reducing undernutrition in children but not for women. For this and other concerns, the NNP was revised few years into its implementation to reflect a lifecycle approach in improving nutrition of women and children, and re-launched in June 2013 [12]. The revised NNP had specific targets of reducing child stunting (from 44.4% to 30%) and undernutrition in women of reproductive age (from 27% to 19%) nationally by 2015. The programme sought to achieve these objectives through strengthening nutrition service delivery within the health sector and nutrition-sensitive interventions across sectors. However, DHS reports in 2014 and 2016 showed these targets were not met for child stunting [4, 13]. This implies the need to strengthen and/or put in practice more comprehensive approaches to solving the problem of undernutrition.

One of such areas is recognizing that health is determined by more than individual factors, such as genetic predispositions and behaviour or life-style choices [14]. One’s social and economic status such as gender, housing conditions, education, income, access to services, age and sanitation, collectively known as *social determinants of health*, are factors that need to be considered [14]. Nutrition is a vital component of health that affects and is affected by these factors. Women and young children are among the most at risk for poor nutrition particularly in Ethiopia where economic and social disparities tend to be greater. Even when food is available, women tend to be malnourished because of their gender status [15]. Women shoulder “triple roles”, including their biological role of bearing-rearing children (reproductive), productive (farm work) and social (community) responsibilities [16]. These roles add significant work-load on women, increasing their risk for poor nutritional status. Undernutrition in women is also known to increase the risk of poor nutritional status in children [7, 17].

The National Nutrition Program in Ethiopia focused on improving nutrition for women and children through nutrition specific and sensitive activities, such as food security, diet diversification, therapeutic feedings, supplementation and fortification. The non-dietary factors, such as gender and socioeconomic-demographic factors, and their importance as underlying causes of maternal and child undernutrition were not emphasized in the program. Although the United Nations Children’s Fund (UNICEF) conceptual framework of malnutrition outlined the underlying causes, research that examined the effects of such factors on mother-child undernutrition was limited in our study setting [18, 19]. Moreover, the impact of the NNP in improving undernutrition in mothers and children at regional levels have not been adequately assessed either.

The purpose of the current study was (i) to estimate levels of maternal and child undernutrition in selected rural communities of Ethiopia five years into the implementation of the NNP and (ii) to explore, the association of maternal-child undernutrition phenomenon with gender, socioeconomic-demographic factors, access-utilization of health services and nutrition related knowledge/practices of mothers during pregnancy and lactation. We hypothesized that levels of maternal-child undernutrition as well as the socio-economic factors affecting it would differ in the selected rural communities.

## Participants and methods

### Setting, participants and ethics

The baseline data reported here was collected as part of a community-based intervention study in three purposively selected rural *Kebeles* (referred herein as communities). *Kebeles* are the smallest administrative units in the government structure and may contain about 500 households each. Two of the three communities (*Guba-Sherero* and *Holagoba-Kukie)* were selected from rural *Halaba*, a Woreda (~District) in Southern Nations, Nationalities and People’s Region (SNNPR). The Woreda/District is located ~85 kilometers northwest of *Hawassa*, the capital of SNNPR. It is known for growing pepper and pulses, which are considered cash crops for the farmers. The third rural community (*Edo-Qontola*) was selected from *Adami-Tulu-Jido-Kombolcha* (ATJK) District near Zeway town, in the Regional State of Oromiya. It is located about 160 kilometers southeast of Addis Ababa, Ethiopia’s capital. Maize, teff, wheat, barley and different oil seeds are the major crops produced in the district. The area is characterized as dry land with both irrigated and rain-fed crop productions. The study communities were part of a larger Ethiopia-Canada project between Hawassa University (Ethiopia) and the University of Saskatchewan (Canada) that sought to improve agricultural productivity and human health in south Ethiopia [20].

In all the three communities, the study population were mothers and their <5yrs of age children. Hence, the inclusion criteria were households in the community with mothers and children <5yrs of age. The sample size was determined using formula for cross-sectional studies in each community [21]: $n_{o}=\left[Z_{1-\frac{\alpha }{2}}^{2}p(1-p)\right]/d^{2}$ and *n* = *n_o_N*/[*n_o_* + (*N* − 1)] where *n_o_*, *n* stand for sample sizes before and after applying “finite population correction” factor, respectively; Z = 1.96, p = probability of expected prevalence, N = total population of interest and d = margin of error. In this study, prevalence of maternal undernutrition was taken as expected prevalence; p = 0.27 with a margin of error (d) of 5% for calculation of the sample size. The calculation yielded 200 households with mother-child pairs per each community. Adding a 5% contingency, a total of 630 mother-child pairs, (i.e. 413 from the two communities in Halaba and 217 from the third community in Zeway) were required. Sample size in Halaba was higher as two of the selected communities from this district were merged for this analysis. The selection of additional community in Halaba was needed for a subsequent intervention-control study. Selection of individual households was carried out by first obtaining a list of all eligible households (those with mothers and under five children) from the local health post and applying simple random sampling. In this study, a household was defined as one that had a mother and at least one under five years of age child and was served by the local health-post regardless of whether polygamy was practiced or not. Whenever households had more than one eligible child, the youngest was considered. The study was approved by the University of Saskatchewan Behavioral Ethics Board (BEH #12–357) as well as the Regional Health Bureaus of SNNPR and Oromiya. All mothers gave oral consent to participate in the study. Due to the low literacy rate in rural Ethiopia, obtaining written consent was not feasible. Oral consent was the most and culturally appropriate way of obtaining informed consent. The consent information was written in simple and easy-to-understand manner which was also translated into the local language. Consent forms were attached on a coded interview questionnaire for each participant. Female data collectors read and explained to each participant the purpose of the research as outlined in the consent form. Once participants gave oral consent to participate in the study, the data collectors wrote the participants’ full name on the consent form to indicate consent. This was then signed by the Principal Investigator (GE), detached from the interview questionnaire and stored in safe locker. The obtaining of oral consent was approved by the ethics committees. The study was carried out in March-June 2013.

### Data collection, tools, variables

Information on the characteristics of participating households was collected by a questionnaire adapted from previous national and local surveys in the region [22–24]. We collected information on household size (number of usual members of the household), number of children <5yrs of age, marital status, polygamy, education of mothers and their husbands (no formal education = never attended school, primary = grade 1–6; post-primary = above grade 6), household headship, usual occupation, ownership of domestic animals, cultivable land, ownership of various household assets, housing characteristics (roof, floor, window material, number of rooms), sanitation facility, access to drinking water, persons responsible for fetching water and time required to fetch water, women’s access to their own farm land, person in charge of agricultural produce and some other variables.

The questionnaire also contained a section that assessed access and utilization of health/nutrition services as well as dietary habits of mothers during their most recent pregnancy and lactation; these items were adapted from the baseline survey for national nutrition program [22]. Information on access and utilization of health/nutrition services included whether mothers had visited any healthcare facility, type of healthcare facility visited, number of visits, place of delivery of the youngest child, and whether mothers had received iron/folate supplement, as well as if mothers had received any health/nutrition education during/after their most recent pregnancy. Additional questions asked include whether mothers abstained from eating certain foods during pregnancy for cultural reasons including the type of foods avoided this way (food taboos), what their eating pattern was like (same, less, more than usual) during pregnancy and lactation, as well as whether mothers knew what balanced diet was.

From the data collected, we created a set of variables to reflect gender, economic and demographic structures of the study households. Difference in years of formal schooling between husband and wife (i.e. years of formal schooling of husband minus that of wife) was used as a proxy measure for empowerment index for the household. The use of years of formal schooling to measure empowerment is supported in literature [25, 26]. We also used the length of time required to fetch drinking water as a proxy measure to estimate work burden on women. WHO/UNICEF recommend that drinking water should be accessible within 30 minutes of round trip from one’s residence [27]; thus, if time required to fetch water took 30 minutes or longer, this would significantly increase work burden.

Women’s access to own-piece of farm land, control over agricultural produce, and polygamy were also variables used to reflect the gender dimensions of participating households. Likewise, we used information on cultivable land size per household and number of persons per household to calculate physiological density (i.e. the number of persons in the household/unit of cultivable land) and which provided information on how much land was available to produce food for the family. We then placed participating households into two categories (≤8 persons/ha or >8 persons/ha) and explored associations with nutritional status of mothers and children. Ownership of livestock is another important resource for agricultural communities in Ethiopia. We used the number and type of livestock information to calculate Tropical Livestock Units (TLU) for each household and divided the households as having low, average or high TLU. One TLU is estimated as the equivalent of 250 kg livestock [28].

A wealth index (WI) was also developed for each participating household to classify households based on socioeconomic status. To achieve this, we used various assets owned by households and other housing and sanitation related characteristics. The assets include ownership of radio, TV, mobile phone, bicycle, horse/donkey cart, motorcycle, handheld torch and oxen. Housing characteristics include roofing structure (corrugated iron or thatched grass roof), flooring materials (cow dung smeared/cement or mud/earth), presence or absence of windows, crowding (persons per sleeping room ≤5 or ≥6) as well as presence or absence of an improved sanitation facility. The use of asset-based approach to determine households’ socioeconomic status is also common in DHS surveys at national level [1, 29]. It is usually used for poor countries where large proportion of the population does not have regular income. Various methods can be used to weigh each item (in our case all binary variables) and calculate the actual index [30]. Each household received a score of 1 or 0 depending on whether it owned a particular asset. We then weighted each binary variable by the inverse of the proportion of households that owned the particular asset or had the particular characteristics [30]. This method assumes that if assets are owned by just the few, it is an indication that those “few” are wealthier than those that do not own the asset, hence they are given greater weight. After calculating WI for each household, households were grouped into low, medium and high WI categories.

Trained female data collectors (mainly nurses) who fluently spoke the local languages administered the interview questionnaire at participants’ residence. Data collection was supervised by the principal investigator (GE) and B.Sc. nutrition graduates who also spoke the local language fluently. Anthropometric measurements of mothers and their children were carried out at the nearest health facility (health post and health centre), local school campus or outside the local *Kebele* office. On separate dates, participants living near to any one of these locations were invited to attend the anthropometric measurement sessions. Those who could not attend were visited at their residence. One person (GE) conducted all anthropometric measurements for both mothers and children to avoid inter-measurer errors. Child anthropometry included weight (measured to the nearest 10g) using electronic scale (Seca 770, Seca Corporation, Hanover, MD, USA); height (for children ≥24 months of age)/recumbent length (for children <24 months) (measured to the nearest 0.1cm) using adult/infant length/stature measuring board (Perspective Enterprises, Portage, MI, USA); head circumference (measured to the nearest 0.1cm) using a flexible non-stretch tape; mid-upper arm circumference (MUAC) (measured to the nearest 0.1cm) using colored insertion tape for children; and triceps skinfold thickness (measured to the nearest 0.2mm) using skinfold caliper (*Holtain* Ltd, Crymych, United Kingdom). Mothers’ anthropometry included weight (to the nearest 0.1kg), height (to the nearest 0.1cm) and MUAC (to the nearest 0.1cm). All measurements were taken in duplicate and averages were considered when the duplicates were similar. If the values were not similar, a third measure was taken to obtain the average of the two similar values. Standardized procedures were employed when taking body measurements [3, 23]. Birthdates for children were determined from immunization cards while age of mothers was asked verbally.

### Data analysis

Questionnaires were inspected daily and errors or inconsistencies were corrected at the field level. Information from questionnaire was entered in SPSS computer package (IBM SPSS Statistics version 20, IBM Corp., Armonk, NY, USA) and cleaned by running simple frequency distributions. Univariate and bivariate analysis was performed for the descriptive statistics. Bivariate and multiple variable regression analysis were performed to explore associations of gender and socioeconomic-demographic variables with maternal and child undernutrition [i.e. Body Mass Index (BMI) < 18.5 kg/m^2^ and Length- or Height-for-age Z score (LAZ or HAZ) < -2 Standard Deviation (SD), respectively] using *Chi square tests and Multiple Classification Analysis (MCA)*. Only variables that were significant in the bivariate analysis were included in the MCA. Maternal BMI and child LAZ/HAZ as continuous variables served as main outcome variables and results were presented with the associated *Eta* (η) and *Beta* (β) values, indicating the bivariate and multiple variable coefficients of variation, respectively.

WHO Anthro (ver. 3.2.2) 2011 was used to analyse all anthropometric data for children. Mean length- or height-for-age z-score (LAZ/HAZ), weight-for-length/height z-score (WLZ/WHZ), weight-for-age z-score (WAZ), MUAC-for-age z-score, head circumference-for-age z-score, triceps skinfold thickness-for-age z-score (only for children ≥ 3 months) and BMI-for-age z-score were calculated. Differences were tested using *t-test for independent samples*. Prevalence of stunting (LAZ/HAZ <-2SD), wasting (WLZ/WHZ <-2SD) and underweight (WAZ<-2SD) were also calculated. Body measurements from mothers were directly entered in SPSS spreadsheet and average MUAC, weight, height, as well as maternal short stature (as %<145cm in height) were calculated [3]. BMI of non-pregnant mothers and severity of maternal undernutrition were estimated per WHO classification of BMI (i.e. <18.5 kg/m^2^ = underweight, 18.5–24.9 kg/m^2^ = normal and >25 kg/m^2^ = overweight/obese). Since MUAC is relatively stable during pregnancy [31], all mothers in the study (including pregnant ones) were grouped as *undernourished* or *normal* using MUAC <23 cm as a cut-off point.

For comparison purposes and where possible, results were presented along with findings from national/regional studies. Statistical significance was set at a *p*-value of <0.05.

## Results

### Household structure: Socioeconomic and demographic characteristics

Individual and household related background characteristics are presented in Table 1. Similar demographic characteristics were observed in both study areas: No significant difference was observed between the communities in median maternal age (28 yrs), household size (6 persons) and in the proportion of <5yrs of age children per household (*p*>0.05). The average household size reported was larger than the national average. Almost all mothers were married. Polygamy was a common practice, particularly in Halaba where one in four mothers had polygamous husbands compared with one in ten at the national level. Compared with the national report, school attendance was much lower among the women; however, their husbands, particularly in Zeway, attended some primary (Grade 1–6) and post-primary education.

**Table 1:** Socio-demographic characteristics of study participants from two rural communities in Halaba and one rural community in Zeway, Ethiopia^a^.

	Halaba (GS & HK) n = 413	Zeway (EQ) n = 217	National/regional reports ^b^^,^ ^c^
Maternal age, yrs. [median (IQR)]	28 (25–32) ^†^	28 (25–30)	-
Household size [median (IQR)]	6.0 (4.0–7.0) ^†^	6 (4.0–8.0)	4.9
Under 5 children/family (%)			
One	56.2 ^†^	61.8	-
Two or more	43.8 ^†^	38.2	-
Marital status (%)			
Married	98.1	94.9	58.1
Other ^d^	1.9	5.1	14.8
Mothers in polygamous family (%)			
Yes	26.6	18.4	11.6
Mothers’ formal education (%)			
No formal education	80.4	70.5	59.8
Primary (grade 1–6)	16.2	22.6	34.5
Post primary (above grade 6)	3.4	6.9	3.7
Husbands’ formal education (%)			
No formal education	53.8	29.5	35.7
Primary	31.5	44.7	56.3
Post primary	14.7	25.8	8.0
Head of HH (%)			
Husbands	89.6	93	76.8
Women	10.4	7	23.2
Mothers’ usual occupation (%)			
Housewife	93.5	89.4	-
Petty trading	4.1	5.1	-
Others ^e^	2.4	5.5	-
Husband’s occupation (%)			
Farmer	90	89.7	87.9
Other ^e^	10	10.3	10.1
Person responsible for fetching water (%)			
Women (mothers)	69	65	70.7
Husband	5.3	10.1	7.3
Children	21.5	16.1	19.8
Maid and other^f^	4.1	8.8	0.9

Abbreviations: GS = Guba-Sherero, HK = Holagoba-Kukie, EQ = Edo-Qontola; SD = Standard deviation; HH = Household; EDHS = Ethiopian demographic and health survey; EHNRI = Ethiopia health and nutrition research institute

^a^ n = 630

^b^ EDHS [1]

^c^ comparison figures represent rural population

^d^ Divorced, widowed, separated

^e^ Other = civil servant, agricultural laborer, tenant farmer, daily laborer etc.

^f^ Employed/rented donkey cart

^†^ *p*>0.05 (Mann-Whitney test for continuous and *χ*^*2*^ *test* for categorical variables).

Table 1 also shows that male-headship was very prominent in the study areas. Ninety percent of the women were not involved in any income generating activities (most were housewives).

Farming was the main occupation of their husbands. Stand-pipe/public tap and wells were the main sources of water (91–96% of the time) for households. Fetching water was primarily women’s responsibility.

### Maternal and child anthropometry and levels of undernutrition

Tables 2 and 3 present information based on maternal and child anthropometric measurements. Mothers in Halaba were taller (157.2cm) than those in Zeway (155.6cm) (*p*<0.05) but had similar average MUAC as Zeway mothers (25cm) (Table 2). Excluding pregnant mothers and those who had babies within the two months preceding the measurement, the average maternal weight was not different while average BMI was slightly lower in Halaba than Zeway mothers (*p*<0.05). Prevalence of maternal short-stature (≤145cm) was 1% to 5%, comparable to the national prevalence of 4%.

**Table 2:** Anthropometric measurements and associated indices for mothers from rural communities in Halaba and Zeway area, Ethiopia.

	Halaba (GS & HK)	Zeway (EQ)	National/Regional Reports^a^
Average anthropometric scores	n = 341	n = 162	
Height (cm)	157.2 ±5.8 ^b^	155.6 ± 6.4	-
MUAC (cm)	24.6 ±2.4	25.1 ± 3.3	-
	n = 266	n = 142	
Weight (kg) ^c^	50.3 ±6.7	51.3 ± 8.7	-
BMI (kgm^-2^) ^c^	20.3 ±2.1 ^b^	21.2 ± 3.6)	20.2
Maternal stature categories (%)	n = 341	n = 162	
Height < 145 cm	1.2	4.9	3.4
Height 145–150 cm	9.1	10.5	-
Height > 150 cm	89.7	84.6	-
Body mass index ^a^ (kg/m^2^) categories (%)	n = 266	n = 142	
Underweight (BMI < 18.5)	21.8	14.1	26.9
Normal range (BMI 18.5–24.99)	75.6	77.5	67.4
Overweight/obese (BMI > 25.0)	2.6	8.4	5.7
Undernutrition based on MUAC (%)	n = 341	n = 163	
Undernourished (< 23 cm)	27	23.5	-
Normal (≥ 23 cm)	73	76.5	-

Abbreviations: GS = Guba-Sherero; HK = Holagoba-Kukie; EQ = Edo-Qontola; SD = Standard deviation; MUAC = Mid-upper arm circumference; BMI = Body mass index; EDHS = Ethiopia demographic and health survey

^a^ EDHS [1]

^b^ Significant between communities at *p*<0.05 *(independent t test)*

^c^ Excludes i) pregnant mothers, ii) pregnant and lactating mothers and iii) mothers who had babies within the last two months prior to the anthropometric measurement

**Table 3:** Anthropometric measurements and associated indices for <5yrs of age children from rural communities of *Halaba* and *Zeway* areas, Ethiopia.

	Halaba (GS & HK)	Zeway (EQ)	National/ Regional Reports ^a^
Mean (SD) z-scores	n = 355	n = 170	
Length/height-for-age	-2.1 ± 1.6 ^b^	-1.6 ±1.6	-1.7
Weight-for-length/height	-0.5 ± 1.2	-0.32 ± 1	-0.5
Weight-for-age	-1.6 ± 1.4 ^b^	-1.1 ± 1.2	-1.3
BMI-for-age	-0.4 ± 1.2 ^c^	-0.13 (± 1	-
Head circumference (cm)	45.2 ± 3.4 ^b^	47.2 ± 2.7	-
Head circumference-for-age	0 ± 1.2	-0.03 ± 1.2	-
	n = 301 ^d^	n = 165 ^d^	
MUAC (cm)	13.1 ± 1.2 ^c^	13.6 ± 1.3	^-^
MUAC-for-age	-1.5 ± 1.1	-1.4 ± 1	-
TSF thickness (mm)	8.5 ± 1.7	8.3 ± 1.7	-
Triceps skinfold-for-age	0.1 ± 1	-0.03 ± 1	-
Prevalence estimates (%)	n = 355	n = 170	
Stunting (HAZ <-2SD	53.5^e^	41.8	44.4
Wasting (WHZ<-2SD)	10.4 ^e^	4.1	9.7
Underweight (WAZ<-2SD)	36.3 ^e^	21.3	28.7
	n = 315 ^d^	n = 169 ^d^	
MUAC-for-age < -2SD	30.2	24.9	-
Gender disaggregated prevalence of undernutrition (%)	n = 355	n = 170	
Stunting			
Male	52	48.1	46.2
Female	55.1^e^	36.3	42.5
Wasting			
Male	12.8 ^e^	3.8	11.1
Female	8	4.4	8.2
Underweight			
Male	39.7 ^e^	21.8	30.5
Female	33.0 ^e^	20.9	26.8

Abbreviations: GS = Guba-Sherero; HK = Holagoba Kukie; EQ = Edo-Qontola; MUAC = Mid-upper arm circumference; TSF = Triceps skinfold thickness; HAZ = Height-for-age Z-score; WHZ = Weight-for-height Z-score; WAZ = Weight-for-age Z-score; SD = Standard deviation

^a^ EDHS [1]

^b^ and ^c^ Significant between Halaba and Zeway at *p*<0.001 and *p*<0.05, respectively *(independent t- test);*

^d^ Minimum child age for the anthropometric indices in these groups is 3 months

^e^ Significant between Halaba and Zeway at *p*<0.05 *(χ*^*2*^ *test)*

Maternal undernutrition (% BMI <18.5) was 22% in Halaba and 14% in Zeway. Most of the mothers had normal BMI. When MUAC was used to estimate proportion of undernourished mothers (MUAC< 23cm), 27% of Halaba and 24% of Zeway mothers fell in the undernourished category. However, no significant differences were observed between communities in proportion of undernourished mothers using either BMI or MUAC (*p*>0.05).

Mean Z-scores of various anthropometric indicators of Halaba children, particularly the ones measuring height/length-for-age and weight-for-age were as low as -2.1 and -1.6, respectively, and significantly lower than the respective values for Zeway children (*p*<0.001) (Table 3).

Prevalence of undernutrition in <5yrs of age children, particularly stunting (54%) and underweight (36%), were very high in Halaba communities. The respective findings in the Zeway community were 42% and 21%—still very high but significantly lower compared with Halaba children (*p*<0.05). The prevalence estimates for Zeway children were lower than those reported at national level, whereas stunting and underweight in Halaba were 20% and 26%, respectively, higher than the national estimates.

Gender disaggregated prevalence of child undernutrition showed no significant difference in prevalence of stunting, wasting and underweight between males and female children within communities. However, more stunted (55.1%) and underweight (33%) female children resided in Halaba than Zeway (36.3% stunted and 20.9% underweight) (*p*<0.05). Among male children, significantly more wasting and underweight were found in Halaba than Zeway.

### Access and utilization of health services, dietary habits and food taboos during pregnancy and lactation

Table 4 presents findings on access and utilization of health services, dietary habits and food taboos during pregnancy and lactation. During their most recent pregnancy, most mothers attended their antenatal care (ANC) at a Health Post or Health Centre (depending on proximity). Health Post is a component of primary healthcare unit that provides basic preventive/ promotive health services mostly for the rural Ethiopia population whereas Health Centre, also part of primary health care unit, severs as a referral for Health Posts and provides curative services to common diseases.

**Table 4:** Access and utilization of health/nutrition services, and dietary habits during most recent pregnancy of mothers from rural communities of Halaba and Zeway area, Ethiopia^a^.

	Halaba (GS & HK)	Zeway (EQ)	National/Regional reports^b^
ANC facility attendance (%)	n = 413	n = 217	
Didn’t go anywhere	11.4	17.1	-
Health Post (HEW)	39.2	2.3	40.9
Health Center (nurse/midwife)	49.4	78.3	-
Hospitals (Doctor/nurse/ midwife)	0	2.3	48.4
ANC visits during last pregnancy (%)	n = 409	n = 217	
< 4 ANC visits	70.5	64.1	63.7 ^c^
4–5 ANC visits	25.9	35.9	14.0 ^c^
6 and above ANC visits	3.7	0.0	22.3 ^c^
Received iron/folate supplement during last pregnancy (%)	n = 412	n = 217	
Yes	72.6	51.6	15.1 ^c^
Place of delivery of youngest child (%)	n = 412	n = 217	
Own home	90.3	82.0	95.7 ^c^
Health facility	8.9	17.9	4.3 ^c^
Received any health/nutrition education during/after last pregnancy (%)	n = 413	n = 217	
Yes	58.8	23.5	-
Stopped eating certain foods during pregnancy for cultural reasons (%)	n = 412	n = 217	
Yes	61.4 ^d^	18.0	-
Consumption pattern during last pregnancy (%)	n = 411	n = 214	
More than usual	4.6	15.4	9.1 ^c^
Same as usual	19.5	54.7	28.1 ^c^
Less than usual	75.9	29.9	55.4 ^c^
Eating pattern during lactation (%)	n = 410	n = 215	
More than usual	75.6	55.3	-
Same/less than usual	24.4	44.7	-
Knowledge of balanced diet (%)	n = 411	n = 215	
Yes	24.0	58.6	-

Abbreviations: ANC = Antenatal clinic; HEW = Health extension workers; GS = Guba-Sherero, HK = Holagoba Kukie, EQ = Edo-Qontola

^a^ n = 630

^b^ EDHS [1]

^c^ EHNRI [22]

^d^ significant at *p*<0.001 *(χ*^*2*^ *test)*

Higher proportion of Zeway mothers had greater number of ANC visits (4–5 times) but also visited a Health Centre. Hospital use for ANC was very minimal or nonexistent in both Halaba and Zeway. Delivery of babies at a healthcare-facility was very minimal, particularly in Halaba. More mothers in Halaba than Zeway reported receiving iron/folate supplement during pregnancy. However, 77% Zeway and 41% Halaba mothers did not get health/nutrition education before or after delivery of their youngest baby. Food taboos were more common in Halaba (61%) than Zeway (18%) (*p*<0.001). Most common foods mothers avoided during pregnancy were animal source foods such as dairy (including cheese, milk/butter milk, yoghurt, and whey), liver, meat, fish and plant source foods such as banana, avocado, kale, sweet potato, and yam. Majority mothers (96% in Halaba and 85% in Zeway) said they ate the “same as usual” or “less than usual” during their pregnancy. In a follow-up question (data not shown), “poor appetite” and “feeling nauseated/sick” followed by “inability to afford desired food” were mentioned as reasons for not eating more during pregnancy. Whereas, “increased appetite due to lactation” was mentioned as a reason for eating more during lactation. In Halaba, only one in four mothers knew what a balanced diet was compared with two in four in Zeway.

#### Associations of gender, household structure, access and utilization of health services and diet related variables with maternal and child undernutrition

Table 5 presents Chi-square associations of various gender and household structure related variables with maternal undernutrition (% BMI <18.5) and child stunting (% LAZ<-2SD). Empowerment index significantly associated with child stunting (*p*<0.05) but not with maternal undernutrition. Women’s access to their own piece of land did not significantly associate with either maternal or child undernutrition. Slightly more stunted (47%) than non-stunted (43%) children were observed in households where only men controlled agricultural produce, but the difference was not significant. Other gender related variables, such as work-burden of women, presence or absence of polygamy in the home and headship of household, did not show significant association with maternal and child nutritional status.

**Table 5:** Association of gender, household structure and location related variables to maternal undernutrition and child stunting in rural Halaba and Zeway, Ethiopia.

	Maternal undernutrition (BMI<18.5)			Child stunting (HAZ <-2SD)		
	No	Yes	χ2, *p*-value	No	Yes	χ2, *p* value
Empowerment index ^a^	n = 408			n = 525		
< 0 ^b^	41.7	8.3	χ2 = 2.971 *p* = 0.396	24.2	26.9	χ2 = 8.694 *p* = 0.034
0–2	6.4	2.5		5.9	2.5	
3–5	18.6	5.1		12.6	11.6	
> 5	14.2	3.2		7.6	8.8	
Women with access to own land	n = 401			n = 519		
No	58.6	14.2	χ2 = 0.003 *p* = 0.954	38.3	35.1	χ2 = 2.162 *p* = 0.142
Yes	21.9	5.2		11.9	14.6	
Control of farm produce	n = 316			n = 414		
Men	70.3	18.2	χ2 = 1.065 *p* = 0.587	43.7	45.8	χ2 = 7.598 *p* = 0.022
Women	2.9	0.5		2.5	0.4	
Both	7.0	1.1		4.1	3.5	
Work burden of women ^c^	n = 408			n = 525		
Shorter distance (<30 minutes)	23.5	5.2	χ2 = 0.145 *p* = 0.703	15	13.7	χ2 = 0.35 *p* = 0.554
Longer distance (≥30 minutes)	57.4	14.0		35.2	36	
Polygamy	n = 408			n = 525		
No	63.7	14	χ2 = 1.187 *p* = 0.276	38.7	38.9	χ2 = 0.121 *p* = 0.728
Yes	17.2	5.1		11.6	10.9	
Household headship	n = 407			n = 522		
Men	74.2	16.7	χ2 = 1.624 *p* = 0.203	46.4	44.6	χ2 = 0.672 *p* = 0.412
Women or both	6.6	2.5		4	5	
Household size	n = 408			n = 525		
< 6	31.4	9.6	χ2 = 3.28 *p* = .047 ^e^	19	23.8	χ2 = 5.374 *p* = 0.02
≥ 6	49.5	9.6		31.2	25.9	
Physiological density^d^	n = 387			n = 497		
8 or less	49.9	11.6	χ2 = 0.088 *p* = 0.766	34.6	29.4	χ2 = 5.615 *p* = 0.018
>8	30.7	7.8		15.5	20.5	
Household TLU	n = 408			n = 525		
Low (<2.5)	46.3	11.3	χ2 = 0.145 *p* = 0.93	29.7	28.4	χ2 = 2.931 *p* = 0.231
Average (2.5–5)	23.5	5.1		13.3	16	
High (>5)	11	2.7		7.2	5.3	
Wealth index	n = 408			n = 523		
Low (<4)	23.2	5.2	χ2 = 1.612 *p* = 0.447	14.9	14.5	χ2 = 2.867 *p* = 0.239
Medium (4–8)	33.7	9.1		20.3	22.9	
High (>8)	24.4	4.4		15.3	12	
Consumption pattern during pregnancy	n = 406			n = 521		
More than usual	6.7	1.7	χ2 = 0.045 *p* = 0.831	5.6	2.7	χ2 = 5.517 *p* = 0.019
Same/less than usual	74.1	17.5		44.7	47	
Maternal knowledge of balanced diet	n = 405			n = 521		
No	51.4	10.6	χ^2^ = 1.517 *p* = 0.218	31.1	32.2	χ^2^ = 0.364 *p* = 0.546
Yes	29.6	8.4		19	17.7	
Geographic location of households	n = 525			n = 525		
Halaba	51	14.4	χ2 = 3.568 *p* = 0.038 ^e^	31.4	36.2	χ2 = 6.355 *p* = 0.012
Zeway	29.9	4.9		18.9	13.5	

Abbreviation: BMI = Body Mass Index; HAZ = Height-for-age z score; SD = Standard deviation; TLU = Tropical livestock unit

^a^ measured using difference in men-women years of formal schooling as a proxy

^**b**^ indicates households where women had more years of formal schooling than men

^c^ measured using time required for fetching drinking water as a proxy; TLU = Tropical livestock unite

^d^ number of persons in a household per unit of cultivable land

^e^ *p*-value from *Fisher’s exact test*

Having a household size of <6 or ≥6 persons significantly associated with maternal undernutrition (*p*<0.05) and with child stunting (*p*<0.05). Physiological density also significantly associated with child stunting (*p*<0.05), but not with maternal undernutrition. Households’ economic structure variables (i.e. TLU of households and wealth index) did not significantly associate with either outcome. Maternal dietary habit during pregnancy (eating “more than usual” or “same/less than usual”) significantly associated with child stunting (*p*<0.05) and underweight (*p*<0.05), but not with maternal undernutrition. Mothers’ knowledge of balanced diet did not associate with undernutrition in either group. However, residing in Halaba or Zeway significantly associated with both maternal undernutrition (*p<*0.05) and child stunting (*p<*0.05).

Access and utilization of health-nutrition services (i.e. ANC attendance, number of ANC visits, place of delivery, receiving iron-folate supplements or health-nutrition education), food taboos and knowledge of balanced diet did not significantly associate with either maternal undernutrition or child stunting. But abstinence from certain foods during pregnancy (food taboo) significantly associated with child wasting (*p* = 0.024).

#### Multiple variable analysis of determinants in maternal and child undernutrition

In a follow-up multiple variable regression analysis of variables that were significant in the bivariate analysis, geographic location of households, i.e. being in Halaba or Zeway (*p*<0.01) and household size, i.e. <6 or ≥6 members/HH (*p*<0.05), in order of importance, remained independent predicators of maternal BMI after adjustment for other factors in the model (Table 6). The overall model was significant and predicted a small variation in maternal BMI (R^2^ = 0.054, *p*<0.015). Likewise, household size (*p*<0.001), men vs. women control of farm produce (*p*<0.01) and physiological density (*p*<0.05), in order of importance, remained independent predictors of LAZ/HAZ in children while dietary consumption pattern during pregnancy (*p* = 0.056) and geographic location of households (*p* = 0.089) were near significant. The overall model was highly significant and predicted a small variation in LAZ/HAZ of children (R^2^ = 0.074, *p*<0.001).

**Table 6:** Determinants of BMI in mothers and HAZ in children from rural communities of Halaba and Zeway area, Ethiopia.

	Mean BMI of mothers					Mean LAZ/HAZ of children				
	N	Unadjusted	(Eta, η)	Adjusted for Factors	(Beta, β)	N	Unadjusted	(Eta, η)	Adjusted for Factors	(Beta, β)
Empowerment index										
< 0	189	20.4	.105	20.5	.079	252	-1.95	.074	-1.90	.050
0–2	34	21.2		21.0		40	-1.54		-1.70	
3–5	84	20.4		20.3		111	-1.92		-1.96	
> 5	65	20.9		20.8		78	-2.01		-2.03	
Control of farm produce										
Men	329	20.5	.053	20.6	.079	430	-1.99	.156	-1.97	.136**
Women	13	21.3		20.7		14	-0.60		-0.68	
Both	30	20.4		19.8		37	-1.61		-1.80	
Household size										
< 6	154	20.1	.127	20.2	.115*	207	-2.21	.157	-2.24	.175***
≥ 6	218	20.8		20.8		274	-1.70		-1.67	
Physiological density										
8 or less	228	20.5	.030	20.5	.010	307	-1.85	.056	-1.81	.084*
>8	144	20.6		20.6		174	-2.03		-2.10	
Consumption pattern during pregnancy										
More than usual	31	20.8	.024	20.5	.006	38	-1.52	.072	-1.56	.065^a^
Same/less than usual	341	20.5		20.5		443	-1.95		-1.95	
Geographic location of households										
Halaba	243	20.2	.168	20.2	.179**	327	-2.09	.159	-2.01	.086^b^
Zeway	129	21.2		21.2		154	-1.55		-1.72	

Mean BMI of mothers: **R** = 0.233; **R**^**2**^ = 0.054; Grand mean = 20.5; Number of cases = 372; **Model**: *F* (9) = 2.314, *p* <0.015

Mean LAZ/HAZ of children: **R** = 0.273; **R**^**2**^ = 0.074; Grand mean = -1.92; Number of cases = 481; **Model**: *F* (9) = 4.210, *p<*0.001

Abbreviations: BMI = Body Mass Index; HAZ = Height-for-age Z score; η = coefficient for the bivariate association; β = coefficient for the multivariate association

*significant at *p*<0.05

**significant at *p*<0.01

***significant at *p*<0.001

^a^ *p* = 0.056

^b^ *p* = 0.089.

## Discussion

We found an alarming level of child stunting in our study communities–54% in Halaba and 42% in Zeway. The levels of maternal undernutrition ranged from moderate (14%) in Zeway to high (22%) in Halaba communities. We also found that 95% of Halaba and 85% of Zeway mothers reported dietary consumption patterns that were “same as usual” or “less than usual” during their most recent pregnancy compared to times of non-pregnancy or lactation. The practice of food taboos by mothers was up to 61% in Halaba and 18% in Zeway communities. Gender and socioeconomic-demographic factors, such as imbalance of power, physiological density, household size and dietary habits during pregnancy, showed significant associations with maternal and child undernutrition.

Targeting the nutrition of most vulnerable groups, such as pregnant-lactating mothers, adolescent girls, infants and young children, is one of the strategies to break the cycle of chronic undernutrition with emphasis to the first 1000 days of life [6, 7, 32]. In agreement to this, Ethiopia launched a National Nutrition Program (NNP) in 2008. The NNP was later revised with the intent to accelerate progress and achieve the Millennium Development Goals [11, 12]. Reducing stunting from 46% to 37% was set as part of the country’s five years (2010–2015) Growth and Transformation Plan [33]. To deliver the service from NNP, Ethiopia took advantage of the already in place national Health Extension Program implemented to improve access to basic health and nutrition services at grass root levels [34]. Nutrition specific and nutrition sensitive programs have been put in place through initiatives such as community-based nutrition and Agricultural Extensions Programs. As a result of these and other related initiatives, Ethiopia has experienced reduced prevalence of stunting, wasting and underweight as well as low-birth-weight babies over the past few years [35].

Despite these encouraging pro-nutrition environments, our findings indicated existence of undernutrition that is of serious public health concern. The prevalence of maternal undernutrition we reported, though lower than the 27% national prevalence [1], presents a continued challenge for the particular region. Level of maternal undernutrition in Ethiopia in 2011 DHS were nearly twice as high as those reported for neighbouring country of Kenya or Uganda in recent DHS [36, 37]. Maternal undernutrition, along with maternal short-stature, is known to increase obstetric risks and maternal morbidity, even in the presence of adequate medical services [38]. In addition, it is a known risk factor for Intra-Uterine Growth Restriction (IUGR), and subsequent low-birth-weight babies, with increased risk of neonatal mortality or stunting [7, 9, 17]. In the current study, mother-child malnutrition phenomenon (i.e. co-existence of undernourished mother with stunted child in the same household) was observed in 10% and 8% of households in Halaba and Zeway, respectively (data not shown). This means a stunted child was present in nearly 50% or more of the households with undernourished mothers. An insult during fetal or embryonic development, per the theory of fetal programing of chronic diseases, has also been linked to adult-diseases, such as coronary heart disease, hypertension, type-2 diabetes and high cholesterol [8, 39, 40].

The alarmingly high levels of child stunting found in our study are 50% - 80% higher compared with levels in neighbouring countries [36, 37, 41, 42]. The prevalence of underweight (a combined reflection of both stunting and wasting) in Halaba was 70% higher than the level found in Zeway community. Both stunting and underweight levels for Halaba children were not only higher compared with values reported in 2011 DHS at national level [1] but also higher than values from DHS 2005. Regardless of what the baseline prevalence in 2005 may have been in these communities, the levels reported here signal a serious warning for nutrition program implementers in the region. Research has already established that stunting compromises both the physical and mental productivity of a country’s future labor force [10, 43, 44]; hence the level of stunting reported here (over 50%) should raise a serious concern for future labor force in the region.

The fact wasting was relatively low (at least, in Zeway community, 4%) indicates that stunting was major contributor to the observed high or very high level of underweight among children. Stunting reflects poor nutrition/care suffered for a long period of time as opposed to wasting, which reflects acute situation of poor nutrition from primary or secondary causes. Addressing wasting in children need not be neglected; but health and nutrition programs need to be specific enough to address stunting. Previous similar studies in younger children (<2yrs or 3yrs of age) from adjacent regions of south Ethiopia also reported high levels of child stunting but not as high as what we reported here [23, 45, 46]. High levels of stunting (31%) were also reported among adolescent girls in Halaba area in a recent community-based study [24].

The lack of significant difference between boys and girls in the gender disaggregated data for stunting, wasting and underweight may indicate that both boys and girls were equally vulnerable to poor nutrition at early age and there was no differential vulnerability based on gender (result not shown). However, stunting tended to be higher (by 12%) in boys than girls in the Zeway community (*p* = 0.08).

Poor nourishment and/or health may be the obvious causes of maternal and child undernutrition. However, women and young children are surrounded by a host of underlying and basic factors in their physical and social environment that play key role in their nutrition and health. These factors, often referred to as *social determinants of health* [14], may greatly impact the growing environment of children. Access and utilization of health/nutrition services may affect maternal and child nutritional status as most mothers in Ethiopia live in rural areas where information/services on nutrition/health are limited. Children are more vulnerable to effects of sub-optimal growing environment because they depend on adults, mostly women, for necessary care. Hence, the adequacy of care women provide to children is affected by their own socioeconomic status—gender related factors, such as their level of empowerment, access-control of important resources and the work-burden they shoulder. In addition, household level factors such as ‘number of people in the household’, wealth, and ownership of livestock are also factors capable of modifying health/nutrition environments for children and women. The socioeconomic and demographic characteristics we presented in Table 1 (such as, low literacy levels, patriarchal family structure, farming as main livelihood and large household size) follow similar trends as in DHS and other local studies [1, 22, 45, 47].

Many of these factors, presented in Table 5, did not show significant associations with maternal nutritional status. However, household size (number of persons in the house) and location of households (Halaba vs. Zeway) were independent predictors both in the bivariate and regression analysis. Larger family size may exacerbate the pressure on women as they carry out their biological role of bearing and rearing children. Empowerment index (a gender variable estimated through the proxy variable “difference in formal schooling between husband and wife”) significantly associated with child stunting in the bivariate but not in the multiple variable analysis. The proportion of stunted children was the smallest (2.5%) and mean LAZ/HAZ was the least (-1.7) in households where the difference in empowerment was the least. This might imply that imbalance in empowerment affects decision making and communications in the household, which in turn negatively influence child-caring practices. In addition, child LAZ/HAZ mean scores where better in households where women (or both gender) controlled farm produce, indicating the importance of ensuring women’s control of important resources to better position them for improved child care.

Likewise, high physiological density may mean less food produced to feed members of the household, hence implication to child nutritional status. Either or both large family size and smaller farm-land holding contribute to high physiological density. This challenge can be mitigated by population policy (i.e., improved reproductive health services to limit unintended pregnancies) and a strong agriculture policy that improves productivity in a small plot of land. Both physiological-density and household size have shown significant association with markers of child stunting in bivariate and multiple variable regression analysis in our study.

Dietary consumption pattern during most recent pregnancy was another important variable that showed significant association with child stunting in bivariate analysis. This association was weaker when LAZ/HAZ was used as continuous outcome variable in the MCA model but was nearly significant. Diet during pregnancy affects maternal health and child development at early stages. Earlier studies looked into factors dictating human growth process in infancy, childhood and puberty, and documented that growth in infancy and early childhood is ‘nutrition-driven’ while growth in the later stages is “hormone-driven”[48]. Some also added that growth at the early stage of infancy is simply “a post-natal continuation of fetal growth” [49]. Maternal undernutrition and poor dietary habit during pregnancy was a known risk factor for intrauterine growth restriction and poor pregnancy outcome (low-birth-weight), both of which were shown to be major risk factors for childhood stunting [7, 50–53]. Besides abstaining from consumption of certain nutritious foods during pregnancy, a large majority (85% -95%) of mothers in our study reported consuming “as usual” or “less than usual” during this critical period (Table 4). Our finding on poor dietary practices during pregnancy was not only consistent with the national report in 2009 [22] but also was an indication to the lack of improvement five years after the launch of the NNP. Though we were unable to connect poor diet during pregnancy and birth-weight in our communities (as ~90% birth happened at home), the alarming levels of child stunting and moderate to high levels of maternal undernutrition indicate the poor growing environment available for the children to thrive. These findings again call for more focused nutrition/health services to and improved utilization by mothers and children in our study communities and similar settings if the country is to sustain progress in the reduction of undernutrition toward achieving the new sustainable development goals [54].

The strength of this study was that it investigated mother-child undernutrition levels five years into the implementation of the NNP phase-1 and explored a set of gender specific or gender sensitive, as well as socioeconomic-demographic variables affecting maternal and child undernutrition in rural Ethiopia. Selection of the study communities was purposive which may limit the external validity of the findings; however, applying our results to other rural communities, given the similarities in health and agriculture services, physical infrastructure, local government etc., could still be applicable and warranted.

## Conclusion

In this study, we have reported very high levels of child stunting (up to 54%) and underweight (up to 36%). We also report moderate to high levels of maternal undernutrition. Gender, as well as socioeconomic and demographic factors, such as empowerment imbalance, control of farm produce, physiological density, household size and dietary habits during pregnancy, showed significant associations, or trends, with maternal and child undernutrition in bivariate and multiple variable analysis. We were also concerned with the poor dietary habits, including existing food taboos, of the women during pregnancy. Our findings stress the need for health program implementers to provide nutrition education before, during and after pregnancy, as the health of mothers is critical to the health of both the mother and fetus. Overall, this cross-sectional study provides important information to nutrition/health service providers in rural communities in evaluating and strengthening existing health programs targeting women and children, and in the development of new programs. Attention should be focussed on women’s education, access and control of resources, as well as better reproductive health services, to improve women’s balance of power in the household and ensure optimum family size for better nutrition.

## Supporting information

S1 Data Anonymized raw data_PONE-D-16-47012R2_FTC. (XLSX)

## References

[1] Central Statistical Agency (CSA) & ICF International. Ethiopia Demographic and Health Survey 2011. Addis Ababa, Ethiopia & Calverton, MD: Central Statistical Agency & ICF International; 2012.

[2] Central Statistical Agency and ORC Marco. Ethiopian Demographic and Health Survey 2005. Addis Ababa, Ethiopia & Calverton, MD: Central Statistical Agency and ICF International; 2006.

[3] WHO Expert Committee on Physical Status. Physical status: The use and interpretation of anthropometry. Report of a WHO Expert Committee. Geneva, Switzerland: World Health Organization (WHO); 1995. 451 p. 8594834

[4] Central Statistical Agency. Ethiopia Mini Demographic and Health Survey 2014. Addis Ababa, Ethiopia: Central Statistical Agency; 2014. 111 p.

[5] CSA and ORC Macro. Ethiopia Demographic and Health Survey 2000. Addis Ababa, Ethiopia & Calverton, MD: Central Statistical Agency and ICF International; 2001.

[6] Black RE, Victora CG, Walker SP, Bhutta ZA, Christian P, de Onis M, et al Maternal and child undernutrition and overweight in low-income and middle-income countries. Lancet. 2013;382(9890):427–51. 10.1016/S0140-6736(13)60937-X 23746772

[7] Black RE, Allen LH, Bhutta ZA, Caulfield LE, de Onis M, Ezzati M, et al Maternal and child undernutrition: global and regional exposures and health consequences. Lancet. 2008;371(9608):243–60. 10.1016/S0140-6736(07)61690-0 18207566

[8] Godfrey KM, Barker DJ. Fetal programming and adult health. Public Health Nutr. 2001;4(2B):611–24. 11683554 10.1079/phn2001145

[9] Kramer MS. Determinants of low birth weight: methodological assessment and meta-analysis. Bull World Health Organ. 1987;65(5):663–737. 3322602 PMC2491072

[10] Grantham-McGregor S, Cheung YB, Cueto S, Glewwe P, Richter L, Strupp B, et al Developmental potential in the first 5 years for children in developing countries. Lancet. 2007;369(9555):60–70. 10.1016/S0140-6736(07)60032-4 17208643 PMC2270351

[11] Ethiopia Federal MoH. Program Implementation Manual of National Nutrition Program (NNP)–I July 2008 –June 2010. Addis Ababa, Ethiopia: Federal Ministry of Health (MoH); 2008.

[12] FDRE. National Nutrition Programme June 2013-June 2015. Addis Ababa, Ethiopia: Federal Democratic Republic of Ethiopia (FDRE),; 2013 p. 45.

[13] Central Statistical Agency (CSA) [Ethiopia] and ICF. Ethiopia Demographic and Health Survey 2016: Key Indicators Report Addis Ababa, Ethiopia and Rockville, Maryland, USA: CSA and ICF; 2016.

[14] Commission on Social Determinants of Health. Closing the gap in a generation: Health equity through action on the social determinants of health. Final report of the Commission on Social Determinants of Health. Geneva, Switzerland World Health Organization; 2008.

[15] FAO. Gender and nutrition: Draft. Food and Agriculture Organization of the United Nations; 2012.

[16] Moser CON. Gender planning in the third world: Meeting practical and strategic gender needs. World Development. 1989;17(11):1799–825.

[17] Kramer MS. Intrauterine growth and gestational duration determinants. Pediatrics. 1987;80(4):502–11. 3658568

[18] Negash C, Whiting SJ, Henry CJ, Belachew T, Hailemariam TG. Association between maternal and child nutritional status in Hula, rural southern Ethiopia: A Cross sectional study. PLoS One. 2015;10(11):e0142301 10.1371/journal.pone.0142301 26588687 PMC4654505

[19] Regassa N, Stoecker BJ. Contextual risk factors for maternal malnutrition in a food-insecure zone in southern Ethiopia. J Biosoc Sci. 2012;44(5):537–48. 10.1017/S002193201200017X 22716940

[20] Hawassa University, University of Saskatchewan. Improving Food Security in the Highlands of Ethiopia throughImproved and Sustainable Agricultural Productivityand Human Nutrition Final Technical Report. 2013 28 Feb. 2013. Contract No.: 106305–001 & 106305–002.

[21] Charan J, Biswas T. How to calculate sample size for different study designs in medical research? Indian J Psychol Med. 2013;35(2):121–6. 10.4103/0253-7176.116232 24049221 PMC3775042

[22] Ethiopian Health Nutrition Research Institute (EHNRI). Nutrition baseline survey report for the National Nutrition Program of Ethiopia. EHNRI; 2010.

[23] Tessema M, Belachew T, Ersino G. Feeding patterns and stunting during early childhood in rural communities of Sidama, South Ethiopia. Pan Afr Med J. 2013;14:75 doi: 10.11604/pamj.2013.14.75.1630 23646211 10.11604/pamj.2013.14.75.1630 PMC3641921

[24] Roba AC, Gabriel-Micheal K, Zello GA, Jaffe J, Whiting SJ, Henry CJ. A low pulse food intake may contribute to the poor nutritional status and low dietary intakes of adolescent girls in rural southern ethiopia. Ecol Food Nutr. 2015;54(3):240–54. 10.1080/03670244.2014.974593 25602600

[25] Guha-Khasnobis B, Hazarika G. Women's status and children's food security in Pakistan—WIDER Discussion Papers 2006. Report No.: 2006/03 Contract No.: ISBN 9291908312.

[26] Groot W, van den Brink HM. Age and education difference in marriages and their effects on life satisfaction. Journal of Happiness Studies. 2002;3:153–65.

[27] WHO & UNICEF. Progress on sanitation and drinking-water: 2010 update France: World Health Organization and UNICEF; 2010.

[28] Teshome G, Kassa B, Emana B, Haji J. Patterns and Determinates of Farm Households’ Investment in Rural Ethiopia: The Case of East Hararghe Zone, Oromia National Regional State. American Journal of Economics. 2013;3(4):191–8.

[29] Dekker M. Estimating wealth effects without expenditure data: Evidence from rural Ethiopia. Ethiopian Journal of Economics. 2006;15(1):35–54.

[30] Howe LD, Hargreaves JR, Huttly SR. Issues in the construction of wealth indices for the measurement of socio-economic position in low-income countries. Emerg Themes Epidemiol. 2008;5:3 10.1186/1742-7622-5-3 18234082 PMC2248177

[31] Gibson RS. Principles of nutritional assessment Second ed. New York, NY: Oxford University Press, Inc.; 2005.

[32] Bhutta ZA, Das JK, Rizvi A, Gaffey MF, Walker N, Horton S, et al Evidence-based interventions for improvement of maternal and child nutrition: what can be done and at what cost? Lancet. 2013;382(9890):452–77. 10.1016/S0140-6736(13)60996-4 23746776

[33] Ministry of Finance and Economic Development. Growth and transformation plan 2010/11-2014/15: Volume II Policy matrix Addis Ababa, Ethiopia: Federal Democratic Republic of Ethiopia; 2010 p. 38.

[34] Banteyerga H. Ethiopia’s Health Extension Program: Improving Health through Community Involvement. MEDICC Rev. 2011;13(3):46–9. 21778960 10.37757/MR2011V13.N3.11

[35] African Union Commission, NEPAD Planning and Coordinating Agency, UN Economic Commission for Africa, UN World Food Programme. The cost of hunger in Africa: Social and economic impact of child undernutrition in Egypt, Ethiopia, Swaziland and Uganda. Addis Ababa, Ethiopia: UN Economic Commission for Africa; 2014.

[36] Uganda Bureau of Statistics (UBOS), ICF International Inc. Uganda Demographic and Health Survey 2011. Kampala, Uganda & Calverton, MD: UBOS and ICF Internaltional Inc.; 2012.

[37] Kenya National Bureau of Statistics (KNBS), ICF Macro. Kenya Demographic and Health Survey 2008–09. Nairobi, Kenya & Calverton, MD: KNBS and ICF Macro; 2010.

[38] Villar J, Valladares E, Wojdyla D, Zavaleta N, Carroli G, Velazco A, et al Caesarean delivery rates and pregnancy outcomes: the 2005 WHO global survey on maternal and perinatal health in Latin America. Lancet. 2006;367(9525):1819–29. 10.1016/S0140-6736(06)68704-7 16753484

[39] Miese-Looy G, Rollings-Scattergood J, Yeung A. Long-term health consequences of poor nutrition during pregnancy. Studies by Undergraduate Researchers at Guelph [Internet]. 2008; 1(2). Available from: https://journal.lib.uoguelph.ca/index.php/surg/rt/printerFriendly/421/666.

[40] Godfrey KM, Barker DJ. Fetal nutrition and adult disease. Am J Clin Nutr. 2000;71(5 Suppl):1344S–52S. 10799412 10.1093/ajcn/71.5.1344s

[41] Kenya National Bureau of Statistics, Ministry of Health, National AIDS Control Council, Kenya Medical Research Institute, National Council for Population and Development. Kenya Demographic and Health Survey 2014: Key Indicators. Nairobi, Kenya: Kenya National Bureau of Statistics; 2015. 65 p.

[42] Matanda DJ, Mittelmark MB, Kigaru DM. Child undernutrition in Kenya: trend analyses from 1993 to 2008–09. BMC Pediatr. 2014;14:5 10.1186/1471-2431-14-5 24410931 PMC3898409

[43] Victora CG, Adair L, Fall C, Hallal PC, Martorell R, Richter L, et al Maternal and child undernutrition: consequences for adult health and human capital. Lancet. 2008;371(9609):340–57. 10.1016/S0140-6736(07)61692-4 18206223 PMC2258311

[44] Black MM, editor The Impact of Nutrition on Cognition from Infancy through School-Age. Nutrition and Cognition in Children: Unilever Pre-Congress Workshop, 18th International Nutrition Congress; 2005 September 19; Durban, South Africa. Vlaardingen, The Netherlands: Unilever Food and Health Research Institute; 2006.

[45] Gibson RS, Abebe Y, Hambidge KM, Arbide I, Teshome A, Stoecker BJ. Inadequate feeding practices and impaired growth among children from subsistence farming households in Sidama, Southern Ethiopia. Maternal and Child Nutrition. 2009;5(3):260–75. 10.1111/j.1740-8709.2008.00179.x 20572929 PMC6860599

[46] Kebebu A, Whiting SJ, Dahl WJ, Henry CJ, Abegaz K. Formulation of a complementary food fortified with broad beans (Vicia faba) in southern Ethiopia. African J of Food, Agriculture, Nutrition and Development. 2013;13(3):7789–803.

[47] Regassa N, Stoecker BJ. Household food insecurity and hunger among households in Sidama district, southern Ethiopia. Public Health Nutr. 2012;15(7):1276–83. 10.1017/S1368980011003119 22152760

[48] Tse WY, Hindmarsh PC, Brook CG. The infancy-childhood-puberty model of growth: clinical aspects. Acta paediatrica ScandinavicaSupplement. 1989;356:38–43; discussion 4–5. 10.1111/j.1651-2227.1989.tb11238.x 2683573

[49] Karlberg J. A biologically-oriented mathematical model (ICP) for human growth. Acta Paediatr Scand Suppl. 1989;350:70–94. 2801108 10.1111/j.1651-2227.1989.tb11199.x

[50] Bhutta ZA, Ahmed T, Black RE, Cousens S, Dewey K, Giugliani E, et al What works? Interventions for maternal and child undernutrition and survival. Lancet. 2008;371(9610):417–40. 10.1016/S0140-6736(07)61693-6 18206226

[51] Kramer MS, Kakuma R. Energy and protein intake in pregnancy. Cochrane Database Systematic Review. 2003;4(4):CD000032. 10.1002/14651858.CD000032 14583907

[52] Kind KL, Moore VM, Davies MJ. Diet around conception and during pregnancy—effects on fetal and neonatal outcomes. Reprod Biomed Online. 2006;12(5):532–41. 16790095 10.1016/s1472-6483(10)61178-9

[53] Ota E, Haruna M, Suzuki M, Anh DD, Tho le H, Tam NT, et al Maternal body mass index and gestational weight gain and their association with perinatal outcomes in Viet Nam. Bull World Health Organ. 2011;89(2):127–36. 10.2471/BLT.10.077982 21346924 PMC3040376

[54] Nations United. Sustainable Development Goals: 17 Goals to Transform Our World: United Nations; 2016 Available from: http://www.un.org/sustainabledevelopment/hunger/.

